# Carbon Nanotubes as Multifunctional Supports for Phthalocyanine-Based Electrocatalysts: Advancing Sustainable Energy Conversion and Environmental Applications

**DOI:** 10.3390/ma19142991

**Published:** 2026-07-10

**Authors:** Man Liang, Ao Wang, Minzhang Li, Xin Zhou, Jian Xue

**Affiliations:** 1 Guangdong Provincial Key Laboratory of Green Chemical Product Technology, School of Chemistry & Chemical Engineering, South China University of Technology, No. 381 Wushan Road, Guangzhou 510640, China; 2023022259@m.scnu.edu.cn (M.L.); xinzhou@scut.edu.cn (X.Z.); 2 Guangdong Provincial Key Laboratory of Quantum Engineering and Quantum Materials, Guangdong Engineering Technology Research Center of Efficient Green Energy and Environment Protection Materials, School of Electronic Science and Engineering (School of Microelectronics), South China Normal University, Foshan 528225, China; 3 Department of Materials Physics, ELTE Eötvös Loránd University, P.O. Box 32, H-1518 Budapest, Hungary; aowang@student.elte.hu

**Keywords:** carbon nanotubes, metal phthalocyanines, electrocatalysis

## Abstract

Carbon nanotubes (CNTs) serve as exceptional multifunctional supports for metal phthalocyanine (MPc)-based electrocatalysts, effectively addressing the inherent limitations of molecular catalysts such as poor conductivity and aggregation. This review systematically summarizes the recent advances in engineering the interface between MPcs and CNTs to optimize performance in sustainable energy conversion and environmental remediation. We categorize the engineering strategies into three synergistic dimensions: (1) dispersion and modification engineering, introducing the most direct physical anchoring dispersion strategy via non-covalent interactions and targeted modifications to yield highly active catalysts; (2) chemical bonding engineering, in which robust axial coordination or covalent grafting creates stable, well-defined active sites and prevents leaching; and (3) geometric and spatial engineering, which exploits CNTs’ unique curvature, atomic defects, inner cavities and one-dimensional architecture to induce strain, symmetry breaking, and nanoconfinement, thereby steering reaction pathways or to construct conductive nanocomposites. These strategies highlight that CNTs are not merely passive scaffolds but active regulators that geometrically and electronically modulate MPcs. By balancing molecular dispersion, charge transfer, and mass transport, CNT-supported MPcs exhibit superior activity, selectivity, and stability for critical electrochemical reactions, including the oxygen reduction reaction (ORR), CO_2_ reduction reaction (CO_2_RR), and nitrate reduction reaction (NO_3_RR), demonstrating substantial potential for advancing sustainable energy technologies and environmental applications.

## 1. Introduction

The escalating global energy crisis and the deterioration of environmental quality have necessitated the development of sustainable technologies capable of addressing resource scarcity and simultaneously mitigating pollution [1]. Electrocatalysis has emerged as a pivotal platform, offering a promising pathway to store intermittent renewable energy in the form of chemical bonds while remediating environmental pollutants through electrochemical transformation [2]. The efficacy of electrocatalytic systems is predominantly governed by the performance of the catalysts. To achieve industrial-scale current densities and high selectivity for target products—such as the oxygen reduction reaction (ORR) for energy conversion [3], the reduction in small molecules like CO_2_ (CO_2_RR) [4] and nitrogenous oxyanions (NO_x_RR) for value-added chemical synthesis and the degradation of pollutants [5,6]—catalysts with precisely engineered active sites are imperative. These catalysts must not only facilitate rapid electron transfer but also optimize the adsorption/desorption energetics of reaction intermediates to suppress competing side reactions, most notably the hydrogen evolution reaction (HER) in aqueous media [7].

In this context, metal phthalocyanines (MPcs) have garnered significant attention as highly efficient molecular electrocatalysts. MPcs are renowned for their excellent intrinsic catalytic activity, structural tunability, and high atom utilization efficiency [8]. However, their practical application is often hindered by poor electrical conductivity and a strong tendency to aggregate via π–π stacking, which masks active sites and degrades performance [9]. To overcome these limitations, integrating MPcs with conductive carbon supports has become a prevailing strategy. While various carbon materials like graphene [10,11,12] and carbon black [13] are utilized, carbon nanotubes (CNTs) stand out as exceptional multifunctional supports. CNTs offer distinct advantages over other carbon allotropes, including a unique one-dimensional tubular morphology that facilitates rapid electron transport along the axis, a high specific surface area for maximizing active site exposure, and remarkable mechanical and chemical stability [14]. Furthermore, the synergistic interaction between MPcs and CNTs can modulate the electronic structure of the metal center, enhancing both activity and selectivity.

**Figure 1:**
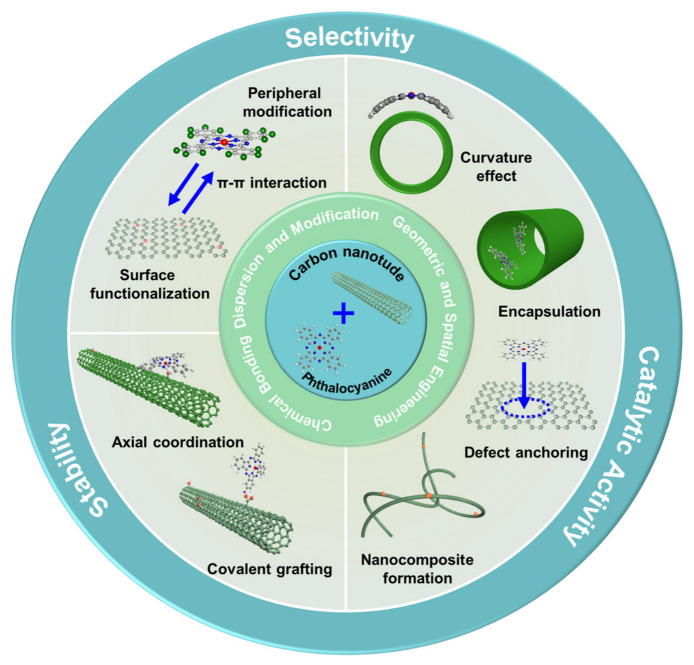
Multiscale engineering approaches for constructing MPc/CNT electrocatalysts.

Despite these advances, the field faces ongoing challenges in balancing catalytic activity, selectivity, and stability. Current reviews often treat carbon supports as passive inert substrates or focus solely on the molecular structure of MPcs, overlooking the critical role of the interface. This review addresses this gap by presenting a paradigm shift: we systematically elucidate the recent advances in engineering the interface between MPcs and CNTs, demonstrating that CNTs are not merely scaffolds but active regulators that geometrically and electronically modulate MPcs’ catalytic site. We categorize the strategies into three synergistic dimensions—(1) dispersion and modification engineering, (2) chemical bonding engineering, and (3) geometric and spatial engineering—providing a comprehensive framework to understand how interfacial interactions dictate catalytic performance. As shown in Figure 1, by linking specific engineering strategies (e.g., axial coordination, curvature-induced strain) to reaction mechanisms and performance metrics, this work offers a practical roadmap for designing next-generation electrocatalysts.

## 2. Structural Characteristics

### 2.1. Metal Phthalocyanines

Metal phthalocyanines (MPcs) are a prominent class of molecular macrocyclic complexes that serve as versatile platforms for electrocatalysis. Their fundamental structure consists of a rigid, planar, and highly conjugated macrocycle formed by four fused isoindole subunits, interconnected via nitrogen atoms at the meso-positions. This architecture creates a central cavity that coordinates a single metal ion in a square-planar M–N_4_ configuration, typically exhibiting D_4_h symmetry. This well-defined, molecularly precise M–N_4_ site is the canonical active center, making MPcs exemplary model systems for studying structure-activity relationships in catalysis [15]. 

A key advantage of MPcs lies in their exceptional synthetic tunability at multiple levels, enabling precise optimization of their electronic and catalytic properties. Primarily, the central metal ion can be varied across a wide range of transition and main-group metals, such as copper (CuPc), cobalt (CoPc), nickel (NiPc), tin (SnPc), iron (FePc), and zinc (ZnPc) [16]. Each metal imparts distinct electronic characteristics to the M–N_4_ site, directly modulating its interaction with reaction intermediates and thus dictating activity and selectivity for different transformations. Beyond the metal center, further fine-tuning is achieved through chemical modification of the organic ligand. Electron-donating or -withdrawing substituents can be introduced at the peripheral (β) or non-peripheral (α) positions of the phthalocyanine ring, systematically altering the electron density at the metal site [17,18].

The molecular nature of MPcs confers significant benefits, including high atom utilization, structurally unambiguous active sites, and the potential for high activity and selectivity that can rival precious-metal-based catalysts at a fraction of the cost [19,20]. These features make them compelling, designable alternatives in sustainable catalysis. However, pristine MPc molecules face intrinsic challenges that limit their practical application: poor electrical conductivity and a strong tendency to aggregate via π–π stacking, which reduces the number of accessible active sites. Therefore, a central research focus is on integrating MPcs with conductive supports like carbon nanotubes to overcome these limitations, creating hybrid materials that combine molecular precision with the robustness required for efficient electrocatalysis.

### 2.2. Carbon Nanotubes

Carbon nanotubes (CNTs) are one-dimensional nanomaterials composed of seamlessly rolled-up graphene sheets, forming hollow cylinders with diameters on the nanometer scale and lengths extending to micrometers or even millimeters, resulting in exceptionally high aspect ratios (10^4^–10^6^) [21]. Their structure arises from the sp^2^ hybridization of carbon atoms, which bond with three neighboring atoms to form a hexagonal lattice. This configuration leads to a delocalized π-electron system across the curved surface, endowing CNTs with remarkable electrical properties. Due to quantum confinement, electrons are restricted to move primarily along the tube’s axis, making individual CNTs either metallic or semiconducting depending on their chirality [22]. Based on the number of concentric graphene cylinders, CNTs are classified into single-walled nanotubes (SWCNTs) and multi-walled nanotubes (MWCNTs). SWCNTs consist of a single graphene cylinder with a typical diameter of 0.4–2 nm, while MWCNTs comprise multiple nested cylinders with interlayer spacings similar to graphite and diameters ranging from 2 to 25 nm or more [23]. This distinct, uniform tubular morphology differentiates CNTs from other carbon allotropes like activated carbon or carbon fibers.

**Figure 2:**
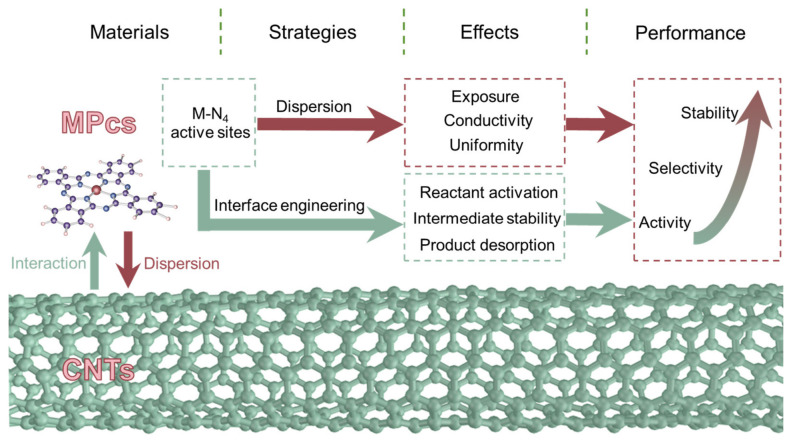
Schematic illustration of the design strategy for MPc/CNT electrocatalysts (from materials to performance).

The unique combination of a high specific surface area, tunable porosity, exceptional thermal conductivity, and mechanical and chemical stability makes CNTs an ideal platform for catalyst support. When used as a support, as shown in Figure 2, CNTs can significantly increase the dispersion and accessible surface area of active catalytic species, such as metal nanoparticles or molecular complexes like MPcs. Furthermore, the strong electronic interaction between the CNT support and the anchored catalytic centers can modulate the electronic structure of the catalyst, often enhancing its activity, selectivity, and long-term stability [24]. This synergy is particularly effective at mitigating common issues like aggregation and low loading of molecular catalysts, positioning CNT-based hybrids at the forefront of advanced electrocatalytic material design.

## 3. Engineering Strategies for CNT-Supported MPcs Electrocatalysts

### 3.1. Dispersion and Modification Engineering

**Figure 3:**
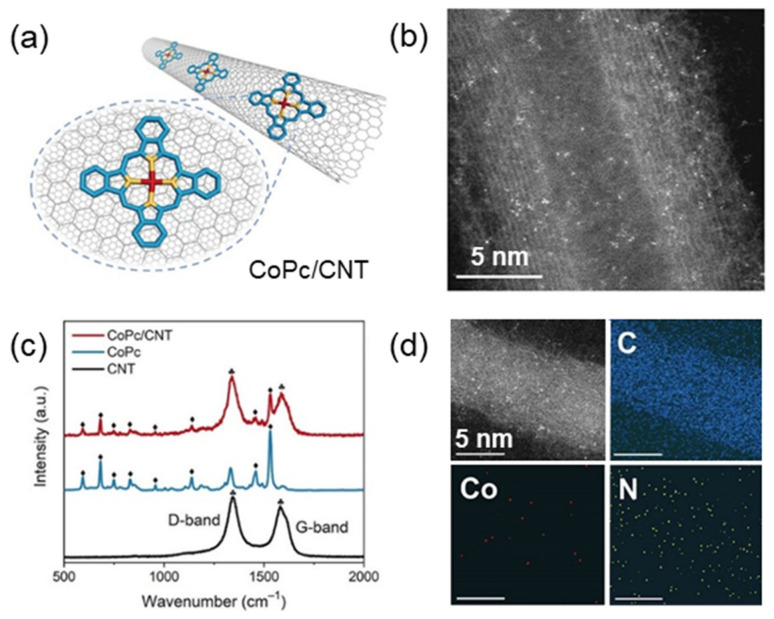
Characterizations of CoPc/CNT catalyst. (**a**) Schematic of the structure of the CoPc/CNT (Red: Co, Yellow: N, Blue: C). (**b**) AC HAADF-STEM image of CoPc/CNT. (**c**) Raman spectra of CoPc/CNT, CoPc, and CNT (Circles denote the characteristic peaks of CoPc, and triangles indicate the CNT). (**d**) EDS mapping of CoPc/CNT. Copyright 2024 by American Chemical Society [28].

Integrating MPcs with highly conductive, high-surface-area CNTs effectively addresses their intrinsic limitations of poor conductivity and molecular aggregation, thereby significantly boosting electrocatalytic activity. The most prevalent strategy leverages non-covalent interactions, mainly π–π interactions and weaker van der Waals forces, to disperse MPcs onto CNTs, which ensures sufficient exposure and stability of active sites while enabling rapid electron transport. This versatile platform has demonstrated remarkable efficacy across a wide array of catalytic reactions. For instance, Jiang et al. [25] reported that anchoring CuPc and CoPc on CNTs creates highly efficient molecular catalysts for nitrogenous oxyanion reduction, with CuPc achieving ~1 A cm^−2^ NH_3_ current density in nitrate reduction reaction (NO_3_RR) and CoPc yielding 466 mA cm^−2^ in nitrite reduction (NO_2_RR), both with >97% faraday efficiency of NH_3_ (FE_NH3_). In the ORR, Lee et al. [26] demonstrated that CNT-supported MPcs exhibit up to 4-fold higher area-normalized activity than those on glassy carbon, with the specific CoPc/CNT catalyst reaching an industrial-scale current density of 200 mA cm^−2^ and 88.7% faraday efficiency of H_2_O_2_ (FE_H2O2_) in a neutral electrolyte. The CoPc/CNT catalyst also excels in organic synthesis and organic-pollutant degradation. Choi et al. [27] utilized it for efficient electrochemical dechlorination of 1,2-dichloroethane to ethylene, attaining near-unity selectivity (FE ≈ 100%), a production rate of 0.56 mmol g^−1^ s^−1^, and >95% pollutant removal in a flow-through reactor. Similarly, Li et al. [28] applied it to the one-step electrochemical oxidation of ethylene to ethylene glycol, achieving a perfect 100% FE of ethylene glycol and a turnover frequency (TOF) of 1.78 min^−1^. As shown in Figure 3, Zhao et al. [29] employed this catalyst for the co-electroreduction of CO and nitrite, securing a total FE exceeding 50% for the synthesis of C–N coupled products like formaloxime and methylamine. Liu et al. [30] employed it to catalyze the C–S coupling of CO_2_ and sulfite, achieving selective electrosynthesis of hydroxymethanesulfonate (HMS) with a FE of 25.7%. These collective results underscore the broad applicability and high performance of the non-covalent interactions-based MPc/CNT electrocatalysts.

Beyond maintaining MPcs dispersion and enhancing electrical conductivity, this physical anchoring strategy serves as the fundamental mechanism for interfacial electronic engineering. The π–π interaction, originating from the inherent electronic compatibility between the sp^2^-hybridized carbon skeleton of CNTs and the conjugated macrocyclic system of MPcs, establishes a conductive bridge for efficient charge transport. This intimate contact facilitates efficient interfacial charge transfer and subtly modulates the electron density of the metal center, thereby optimizing the adsorption energetics of key reaction intermediates. For instance, Feng et al. [31] demonstrated that the CoPc/CNT catalyst, engineered for CO_2_RR under pure acid conditions, stabilized the critical *CO_2_ intermediate via interfacial charge transfer between CoPc and CNTs at the acidic environment of the electrode surface. This effect enabled the catalyst to achieve CO faradaic efficiency (FE_CO_) of 60% in a flow cell and 73% in a membrane electrode assembly (MEA), respectively. Notably, unlike conventional H-cell limited by slow mass transport, flow cells enhance reactant delivery to boost current densities, while MEAs integrate catalyst and electrolyte layers to minimize ohmic losses, making them the preferred platform for industrial-scale implementation.

A refined approach in interfacial engineering focuses on the active modification of the CNTs surface. This strategy aims to create a designed local microenvironment that precisely modulates the properties of anchored MPc molecules, thereby exerting control over catalytic pathways. Harmon et al. [32] demonstrated that the surface chemistry of CNT supports plays a decisive role in steering the catalytic pathway of CoPc. The CoPc supported on pristine CNTs (CoPc/CNT) favored ammonia production with a FE_NH3_ of 70%, while shifting to oxidized CNTs (CoPc/OCNT) diverted the reaction toward HER, exemplifying precise reaction control via support modification. Similarly, Lee et al. [33] reported a supramolecular engineering strategy to optimize CoPc/CNT catalysts for ORR. They employed acidic oxidation to treat the CNTs, introducing a high density (8 at%) of oxygen functional groups, including epoxy (C–O–C), -OH, -COOH, and C=O species, denoted as CNT(O). These groups engage in a reinforced dipole-induced dipole interaction that strengthens the π–π stacking with CoPc, thereby immobilizing the CoPc molecules and preventing their aggregation. This supramolecular interaction modulates the electronic structure, rendering the Co center electron-deficient and optimally tuning the adsorption free energy of the critical *OOH intermediate, which enables the CoPc-CNT(O) catalyst to demonstrate outstanding performance, achieving an industrially relevant current density of 300 mA cm^−2^ with a low overpotential of 280 mV and sustaining stable production for over 100 h in a neutral electrolyte.

In addition to electronic and chemical modifications, the rational design of CNTs extends to the precise control of surface wettability. Yang et al. [34] demonstrated that using graphitized CNTs (GCNT) as a support for vanadyl phthalocyanine (VOPc) served multiple purposes. The graphitic structure not only enhanced conductivity and preserved the structural integrity of the VOPc active center under harsh conditions but also increased surface hydrophobicity, which collectively favored the 2e^−^ ORR pathway by limiting water flooding and enhancing O_2_ access. Consequently, the VOPc/GCNT composite maintained selectivity of >90% FE_H2O2_ even at an ultrahigh current density of 3.5 A cm^−2^.

Complementing strategies for engineering CNT supports, an equally powerful approach involves molecular engineering of the MPcs themselves. While the CNTs establish the foundational conductive scaffold, strategic peripheral functionalization of the MPcs offers a direct pathway to effectively tune the electronic structure of the metal center (M-N_4_), the adsorption energetics of intermediates, and the interfacial properties, providing a direct means to optimize catalytic performance for target reactions. The introduction of charged functional groups serves as a potent strategy to modulate the local electrostatic environment at the catalytic interface. For instance, Wang et al. [35] reported a trimethylammonium, tert-butyl-functionalized CoPc (CoPc2) immobilized on MWCNTs, which achieved a high CO partial current density of 165 mA cm^−2^ with 94% FE_CO_ in a flow cell. This performance enhancement is attributed to the through-space electrostatic interaction between the cationic trimethylammonium moiety and the electron-rich oxygen atoms of CO_2_, which facilitates the reductive coordination and activation of CO_2_ at the Co-N_4_ center. Systematic electronic tuning of the metal center via electron-donating or electron-withdrawing substituents further optimizes the reaction energetics. Zhang et al. [36] demonstrated that a methoxy-substituted NiPc (NiPc-OMe@CNT) attained >99.5% FE_CO_ at current densities up to 300 mA cm^−2^ in 1 M KHCO_3_ electrolyte, owing to the electron-donating methoxy groups stabilizing the key *COOH intermediate via hydrogen bonding during the proton-coupled electron transfer step and thereby suppressing the competing HER. Jiang et al. [37] further demonstrated that this identical NiPc-OMe@CNT catalyst could maintain exceptional performance in acidic media, achieving >98% FE_CO_ at current densities up to 400 mA cm^−2^. They attributed this robustness to the intrinsically high preference of the well-defined Ni-N_4_ active sites for CO_2_RR over HER, combined with the absence of side-reaction sites within the molecularly precise architecture, and the effective suppression of HER by K^+^ ions in the electrolyte. Similarly, Chen et al. [38] illustrated that electron-withdrawing fluorine substituents on SnPc (SnPc–8F@CNT) enabled 80.8% FE for formate (FE_formate_) production with a remarkable partial current density of 600 mA cm^−2^, ascribed to the fluorine atoms modulating the Sn active site electron density to weaken the *OCHO/*HCOOH binding affinity, promote intermediate desorption as formate, and thereby alleviate the kinetic barrier of the rate-limiting step.

**Figure 4:**
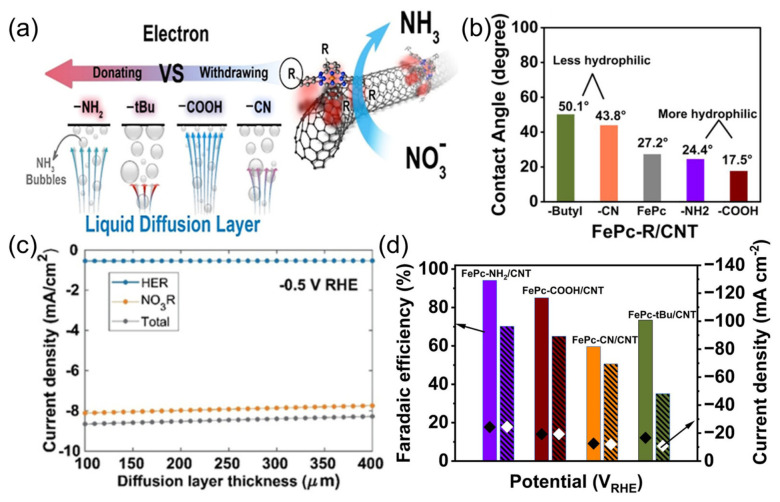
(**a**) Schematic illustration of the relationship between the electronic effect and wettability on NO_3_RR performance. (**b**) Contact angle measurements of the functionalized FePc−R/CNT (R = NH_2_, COOH, CN, t−Bu) catalysts. (**c**) Simulated relationship between the diffusion layer thickness and partial current densities for the HER and NO_3_RR. (**d**) Comparison of the NH_3_ partial current density and FE for each catalyst at −0.6 V vs. RHE, obtained from 30 min chronoamperometry (CA, solid bars) and 6 h CA (striped bars) tests. Copyright 2026 by the American Chemical Society [39].

Furthermore, modifying the MPcs with hydrophilic functional groups represents an effective strategy for tuning interfacial mass transport and electrode wettability. Noor et al. [39] systematically investigated the impact of electronic structure and wettability on the performance of functionalized FePc (R = NH_2_, COOH, CN, t-Bu) supported on CNTs for NO_3_RR. As Figure 4 shows, the study revealed that hydrophilic functional groups directly modulate electrode wettability (contact angles ranging from 17.5° to 50.1°), which in turn governs the thickness of the diffusion layer. Density functional theory (DFT) calculations further revealed that the electron-donating -NH_2_ group elevates the HOMO level of the Fe-N_4_ center, which facilitates the hydrogenation of NH_x_ intermediates. Consequently, the electrode loaded by strongly hydrophilic FePc-NH_2_/CNT catalyst exhibited a thinner diffusion layer, facilitating enhanced NO_3_^−^ accessibility and thereby achieving a maximum FE_NH3_ of 94.1% and a partial current density of 22.9 mA cm^−2^ for NH_3_ production. However, this interfacial advantage diminished rapidly over time; the catalyst selectivity dropped markedly after 6 h of operation, indicating that merely tailoring the molecular structure is insufficient and must be coupled with additional regulation strategies to sustain the diffusion-layer modulation.

Martínez-Sánchez et al. [40] immobilized MPc (M = Fe, Co, Ni, Cu) onto CNT buckypaper (BP) to form self-supported electrodes for CO_2_RR. By introducing N,P-containing polymeric species to modulate the BP surface chemistry, they demonstrated that microenvironment hydrophilicity affects mass transport, enabling tuning of the syngas H_2_/CO ratio. Li et al. [41] demonstrated that constructing a three-dimensional self-supported electrode by growing CNT networks on carbon cloth and anchoring di(ethylene glycol)-substituted CoPc (CoPc-DEG) synergistically enhances mass transport. The integrated porous architecture of the electrode reduces the diffusion layer thickness, while the hydrophilic DEG side chains create a favorable microenvironment for water adsorption; this multiscale engineering enabled the catalyst to operate stably for 25 h at a high current density of 1000 mA cm^−2^ for efficient HER. For non-HER aqueous reactions such as CO_2_RR, excessive hydrophilicity is counterproductive as it intensifies competing HER; a moderately hydrophobic microenvironment is instead required to facilitate gas reactant access and suppress water electrolysis. Moreover, owing to the inherently high porosity of CNTs, gas diffusion layers frequently suffer from severe water flooding and salt precipitation even under laboratory-scale flow cell and MEA tests, necessitating effective hydrophobic treatment of the electrode or hydrophobic modification of the catalyst itself [4]. Qin et al. [42] rationally tuned the microenvironment of a CoPc/CNT catalyst by engineering an amorphous-dominant PTFE modification. This strategy established a robust three-phase interface, enabling a high FE_CO_ of 96% and maintaining >85% FE_CO_ at a current density of 200 mA cm^−2^ in a MEA. Zhou et al. [43] designed an amphiphilic CoPc (TC-CoPc) featuring a hydrophobic macrocycle adsorbed onto MWCNTs and hydrophilic carboxyl side chains extending into the electrolyte, which optimized the electrode/electrolyte interface and improved mass transfer to suppress HER. In a MEA, this catalyst established a built-in water layer by retaining moisture from humid gas, effectively dissolving and migrating carbonate crystals to prevent electrode fouling, achieving a FE_CO_ exceeding 99% at a current density of 50 mA cm^−2^ over a 27 h stability test. This hierarchical design can be extended to the system level, where Shen et al. [44] leveraged the strong *CO_2_ adsorption capability of CoPc/CNT to confine limited CO_2_ molecules at active sites for efficient activation, while implementing a cathodic electrolyte cross-flow strategy to mitigate the OH^−^-induced CO_2_-depletion layer and enhance mass transport. This synergistic approach achieved a high FE_CO_ of 96.2% at industrial-level current densities of 50–300 mA cm^−2^ in neutral bicarbonate media.

Building upon the understanding of electronic coupling, achieving optimal performance further necessitates precise engineering of the MPcs loading amount on the CNTs. This parameter critically dictates whether the catalyst exists as a monolayer of isolated, active molecules or as aggregated clusters, directly impacting both the number of accessible sites and the interfacial environment. An insufficient loading leads to a scarcity of active sites, limiting the overall activity, while an excessive amount often results in inhomogeneous dispersion and molecular aggregation, which masks active sites and diminishes catalytic efficiency. For instance, Wu et al. [45] demonstrated that precise control of the CoPc concentration in the precursor solution is critical to achieving its molecular-level dispersion on CNTs, which enabled a maximum loading of approximately 2.62 wt.% and endowed the catalyst with superior CO_2_-to-CO performance, including a FE_CO_ of 95% and a current density of 200 mA cm^−2^. Conversely, the loading amount can also be a strategic tool to manipulate selectivity by modulating the properties of the support surface itself. Tang et al. [46] applied a ZnPc/CNT catalyst for the selective electrosynthesis of hydroxylamine for NO_3_RR. By increasing the ZnPc loading to 0.9 wt.%, they effectively suppressed the inherent ammonia-producing activity of the bare CNT support, achieving a 53% FE of hydroxylamine with a partial current density exceeding 270 mA cm^−2^. Collectively, these findings establish that loading optimization is not merely about maximizing the number of active molecules, but a delicate balance to simultaneously ensure high dispersion, sufficient site density, and control over the composite’s interfacial chemical properties.

However, MPcs anchored solely via non-covalent interactions are prone to molecular stacking on the support surface. Especially at high loadings, this can obscure a portion of the active sites, thereby limiting their practical catalytic efficiency. To address this issue, Jin et al. [47] investigated the *In Situ* growth of a NiPc supramolecular ribbon on CNTs via auxiliary hydrogen-bonding to stabilize the dispersion. This unique architecture effectively mitigated molecular stacking, enabling the catalyst to achieve >98% FE_CO_ over a wide potential range, a high current density of 353 mA cm^−2^, and stable operation for 40 h in CO_2_RR. Zhu et al. [48] developed a tetra-crown-ether-substituted CoPc that forms a host-guest complex with K^+^ ions and is anchored onto CNTs via simple dip-coating for CO_2_RR. The CNT supports, through π–π interactions and the reinforced electrostatic attraction from the crown-ether-K^+^ complex, enable single-molecular dispersion and a stable catalyst interface, achieving a high TOF of 111 s^−1^ at 38 mA cm^−2^ with >96% FE_CO_ and overcoming the typical trade-off between intrinsic activity and current density. These studies demonstrate that augmenting π–π interactions with directional forces like hydrogen bonding or tailored host-guest electrostatic attractions provides a powerful strategy for advanced interfacial engineering.

### 3.2. Chemical Bonding Engineering

Although π–π interactions effectively mitigate issues of molecular dispersion and conductivity, this strategy is intrinsically constrained. The non-covalent binding is relatively weak, offering limited scope for electronic modulation, and it remains susceptible to molecular aggregation and catalyst leaching under demanding operational conditions. In contrast, constructing well-defined chemical bonds between MPcs and CNTs generates a much stronger interfacial interaction. This robust integration not only more effectively prevents catalyst aggregation and detachment but also establishes a direct electronic conduit, enabling precise and potent modulation of the electronic structure and coordination environment of the metal sites.

**Figure 5:**
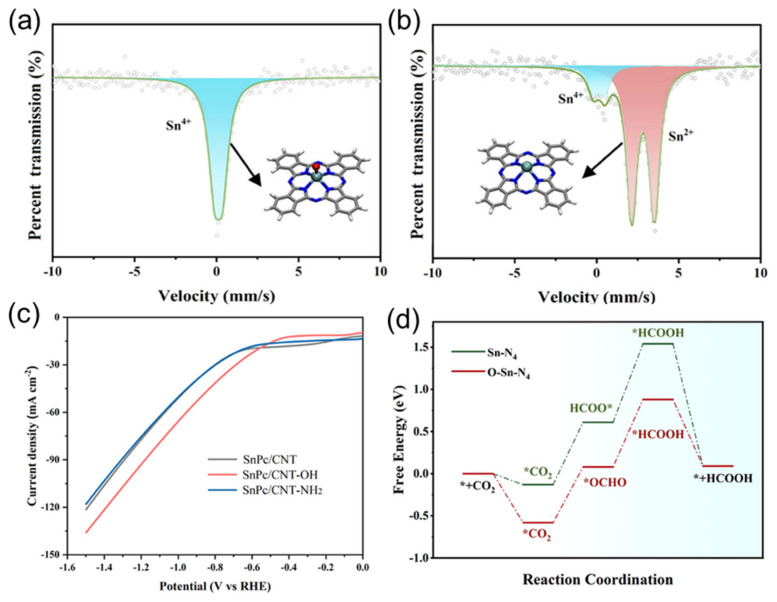
Room-temperature ^119^Sn Mössbauer spectra for (**a**) SnPc/CNT–OH and (**b**) SnPc/CNT, respectively. (**c**) Linear sweep voltammetry curves of SnPc/CNT, SnPc/CNT–OH, and SnPc/CNT–NH_2_ for the CO_2_RR, measured in a flow cell with 1 M KOH electrolyte. (**d**) Calculated Gibbs free energy diagrams for CO_2_RR to HCOOH over O–Sn–N_4_ and Sn–N_4_ sites. Copyright 2023 by the American Chemical Society [51].

Among the primary strategies for chemical bonding engineering, a highly effective approach is the axial coordination engineering, which utilizes functional groups on the CNTs surface as electron donors to form axial coordination bonds with the metal center of MPcs. Importantly, this direct coordination to the metal center not only modulates the electronic structure but also acts as a robust anchoring mechanism, effectively suppressing the leaching of metal sites and significantly enhancing the long-term stability of the catalyst [49,50]. The introduction of axial O-donor ligands, for instance, provides versatile electronic modulation, enabling the stabilization of specific metal oxidation states, fine-tuning of the d-band center, and even manipulation of the metal’s spin state, thereby steering reaction pathways with high selectivity. Deng et al. [51] investigated axial oxygen-coordinated single-Sn-atom sites derived from SnPc molecules anchored on OH-functionalized CNT and found that the axial O-Sn-N_4_ coordination stabilizes Sn(IV) and promotes the formation of the key Sn-O-CO_2_ intermediate, thereby achieving high activity and selectivity for CO_2_RR to formate (FE_formate_ = 89.4%). As illustrated in Figure 5, ^119^Sn Mössbauer spectroscopy definitively confirmed this axial coordination: while commercial SnPc exhibited mixed Sn(II)/Sn(IV) signals, the SnPc/CNT-OH sample showed a single doublet (IS = 0.04 mm s^−1^, QS = 0.50 mm s^−1^) matching DFT-calculated parameters for the O–Sn–N_4_ structure, thereby excluding metallic Sn^0^ or SnO_2_ clusters. Li et al. [52] demonstrated that the axial Co–O coordination in CoPc anchored on oxidized SWCNT downshifts the d-band center of the Co site, optimizing *OOH binding in the ORR process for highly efficient and stable H_2_O_2_ electrosynthesis with a production rate of 3.12 mol g^−1^ h^−1^ over 75 h.

Furthermore, axial oxygen bridges can induce fundamental changes in the spin state of the metal center, unlocking superior intrinsic activity. Zeng et al. [53] demonstrated that covalently anchoring FePc to CNTs via oxygen bridges (FePc-O-CNT) yields a catalyst dominated by low-spin Fe(III) sites. *Operando* spectroscopic studies identified that under CO_2_RR conditions, this structure facilitates the *In Situ* generation of highly active LS Fe(II) sites. The formation of this specific low-spin Fe(II) active site, as opposed to the more common high-spin state, was identified as the key to drastically enhancing the intrinsic activity. This spin-state engineering led to exceptional performance, achieving 99% FE_CO_ with an extremely high TOF of 14.7 s^−1^. Chemical bonding also provides a direct means to redirect reaction pathways. Luo et al. [54] anchored CoPc to hydroxyl-functionalized CNT (CNT-OH) via an axial Co–O coordination bond. This targeted bonding strategy successfully switched the CO_2_ reduction product from CO to methanol, achieving a 32.0% Faradaic efficiency for methanol (FE_CH3OH_) and a partial current density of 18.3 mA cm^−2^.

Axial coordination with nitrogen-donor species, ranging from organic functional groups to heteroatom-doped carbon supports, represents a powerful strategy for modulating the electron density and orbital occupancy of the metal center in supported MPcs [55]. This precise electronic tuning enables the rational enhancement of both catalytic activity and product selectivity. Using amino groups (-NH_2_) as axial ligands enables direct Co–N bond formation. For instance, Li et al. [56] anchored CoPc onto amino-functionalized CNT (NH_2_-CNT) via axial Co–N coordination, which effectively tuned the electronic structure of the Co center, enabling a high CO_2_RR performance with a TOF of 31.4 s^−1^, near 100% FE_CO_ at 225 mA cm^−2^, and stable operation for 100 h. Expanding this strategy to doped CNT supports, Chen et al. [57] demonstrated that CoPc supported on N-doped CNT (CoPc/N-CNT) forms a stable Co–N_5_ axial coordination structure. This engineering changed the rate-determining step of the CO_2_RR, significantly lowering the energy barrier for *COOH formation and suppressing both the HER and further reduction of *CO, thereby achieving a high TOF of 19.2 s^−1^ and over 95% FE_CO_ at a high current density of 800 mA cm^−2^.

The pyridine-functionalized CNT (py-CNT) platform is a particularly versatile and common N-donor. Its coordination capability is highly effective for different metal centers. Zhu et al. [58] reported that axial Co–N bonding between CoPc and py-CNT optimized the electronic structure of Co sites, delivering a record-high TOF of 34.5 s^−1^ at -0.63 V vs. RHE with >98% FE_CO_ for CO_2_RR. Lyu et al. [59] further compared the axial coordination effects using CoPc anchored on aminobenzene (Am), cyanobenzene (Cy), and Py- functionalized CNT (CoPc-Am-CNT, CoPc-Cy-CNT, and CoPc-Py-CNT) for CO_2_RR, demonstrating that the electron-donating Am ligand induced the strongest Co–N bonding and shifted the Co d-band center closer to the Fermi level, resulting in a high TOF of 5.3 s^−1^; whereas the electron-withdrawing Py and Cy ligands facilitated a more positive shift in the Co^2+^/Co^+^ potential, achieving superior FE_CO_ (>90%) at lower overpotentials. This aryl-substituted group strategy is also highly effective for coordinating with FePc, creating a well-defined FeN_5_ active site. Xu et al. [60] demonstrated that this axial coordination strategy for FePc not only broke molecular symmetry and optimized the Fe center’s electronic structure but also facilitated interfacial charge transfer [24]. Consequently, it doubled the NH_3_ yield to 21.7 μg mg_cat_^−1^ h^−1^ and the FE_NH3_ to 22.2% for the electrocatalytic nitrogen reduction reaction (NRR) compared to a conventional π–π stacked counterpart. Wang et al. [61] demonstrated the outstanding durability of the same axially coordinated FePc/py-CNT catalyst in the NO_3_RR. The catalyst sustained a high FE_NH3_ (>90%) over 15 consecutive cycles and maintained stable performance during a continuous 120 h electrolysis at 1 A cm^−2^. Post-reaction characterization confirmed the preservation of the catalyst’s structural integrity, underscoring the effectiveness of the axial coordination strategy in constructing not only highly active but also exceptionally robust catalytic sites.

Beyond the prevalent O- and N-donor ligands, the toolbox for axial coordination engineering is remarkably diverse. Halogen ligands, for instance, are effective in inducing symmetry breaking at the active site. Liu et al. [62] developed a “Cl-mediation” strategy to anchor FePc on CNTs via axial Cl coordination. This Cl ligand successfully transformed the local symmetry of FePc from D_4_h to C_4_v and optimized the electronic structure of the Fe-N_4_ center, leading to significantly enhanced ORR activity with a half-wave potential of 0.91 V and a Tafel slope of 33.23 mV dec^−1^. This axial coordination was confirmed by XPS and EXAFS, which identified the formation of Fe-Cl bonds and a distinct Fe-Cl scattering path. Crucially, electron paramagnetic resonance (EPR) spectroscopy revealed a significant increase in unpaired electrons upon Cl introduction, providing direct electronic evidence for the disruption of the planar symmetry.

Cao et al. [63] designed a series of axially X-coordinated CoPc/XCNT(X=O, N, S) for the electrocatalytic semi-hydrogenation of acetylene to ethylene, with the optimal CoPc/NCNT achieving 86.7% FE of ethylene and a TOF of 117.0 s^−1^ at 500 mA cm^−2^, where the axial nitrogen coordination from the CNT support electronically modulates the Co center to optimize reactive hydrogen (*H) adsorption and utilization. Mustapha et al. [64] loaded NiPc onto boron-doped CNT (NiPc/B-CNT) for the CO_2_RR, where the electron-deficient B dopant uniquely alleviated Ni electron depletion by suppressing interfacial electron transfer to the support, thereby optimizing *COOH adsorption and achieving a high FE_CO_ of >98% at current densities from 20 to 80 mA cm^−2^ with 37 h of robust stability. Furthermore, metal-to-metal bonding or intermetallic coupling can be leveraged to create unique active sites. Lv et al. [65] constructed a molecular-atomic coupled catalyst by integrating FePc with Fe-N_4_ sites anchored on nitrogen-doped CNT (FePc-Fe-NCNT). The strong electronic coupling between FePc and the Fe-N_4_ sites on the CNT support triggered a spin-state transition from low-spin to intermediate-spin at the Fe center, which optimized the adsorption/desorption of oxygen intermediates and enabled an exceptional ORR half-wave potential of 0.89 V with negligible degradation after 10,000 cycles. The axial coordination strategy is not limited to substrate modification but can be directly applied to the MPc molecule itself; specifically, Liu et al. [66] anchored axial phosphate (PO_4_) groups onto CoPc supported by CNTs (P-CoPc@CNT) to construct a tailored Co-N_4_ active center. They discovered that the axial PO_4_ ligand directly participates in the OER cycle by transforming into an HPO_4_-Co-N_4_ intermediate, which replaces the traditional rate-limiting Co-O-H dehydrogenation with a lower-energy HPO_4_ dehydrogenation step, thereby achieving a low overpotential of 300 mV and a Tafel slope of 41.7 mV dec^−1^ at a low Co loading (2.7%).

**Figure 6:**
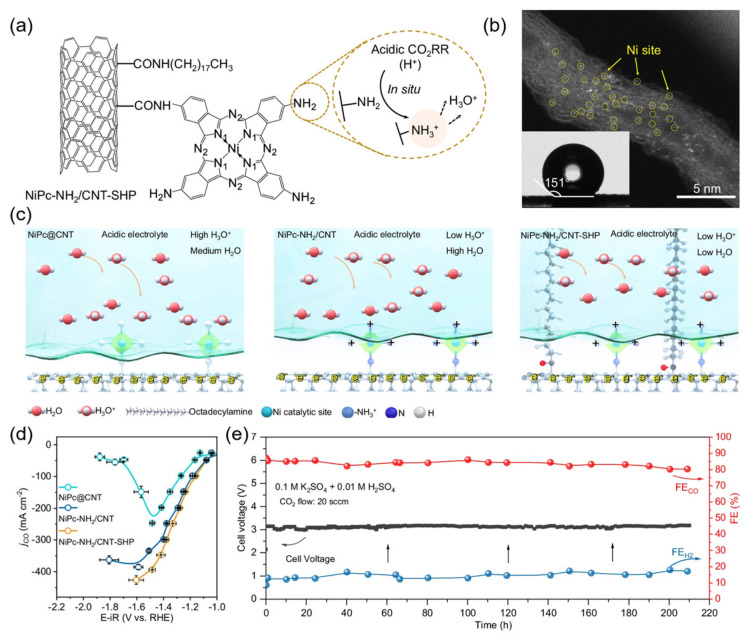
(**a**) Mechanism diagram of the NiPc−NH_2_/CNT−SHP catalyst. (**b**) AC HAADF−STEM image of NiPc−NH_2_/CNT−SHP, with the inset showing contact angles of water. (**c**) Schematic presentation of water species distribution on the surface of NiPc@CNT, NiPc−NH_2_/CNT, and NiPc−NH_2_/CNT−SHP. (**d**) j_CO_ of NiPc−NH_2_/CNT−SHP, NiPc-NH_2_/CNT and NiPc@CNT in 0.5 M K_2_SO_4_ + H_2_SO_4_ (pH = 1). (**e**) The chronopotentiometry stability test of the acid PSE reactor at the current density of 100 mA cm^−2^ and the corresponding FE_CO_, black arrow: renewing electrolyte. Copyright 2025 by Springer Nature [71].

In addition to axial coordination, covalent grafting offers an alternative and robust strategy for integrating MPc molecules with CNT supports [67,68]. This approach involves forming direct covalent bonds between peripheral functional groups on the phthalocyanine macrocycle and complementary groups on the CNT surface. The strong, directional nature of covalent bonds ensures intimate contact and efficient electronic communication between the molecular catalyst and the conductive scaffold, which can lead to enhanced performance in electrocatalysis. Crucially, as shown in Table 1, this vertical covalent linkage not only effectively suppresses the leaching of entire MPc macrocycles from the support but also introduces significant steric hindrance, preventing the π–π stacking-induced aggregation of MPc molecules and thereby preserving the accessibility of active sites. Su et al. [69] developed an *In Situ* covalent grafting strategy to immobilize cationic CoPc (CoTMAPc) onto CNTs via a diazo-reaction. The resulting catalyst achieves an industrially relevant current density of 239 mA cm^−2^ with 95.6% FE_CO_ and a high TOF of 102.9 s^−1^ in CO_2_RR. The covalent grafting strategy effectively prevents catalyst leaching and stabilizes the interface, while the electron-withdrawing quaternary ammonium groups optimize the Co-N_4_ center’s electronic structure to favor *COOH formation and *CO desorption, thereby enhancing both activity and durability. Xiong et al. [70] covalently anchored amino-substituted CoPc (CoPc-NH_2_) and hydrophobic octadecylamine (ODA) onto CNTs to create atomically dispersed active sites with precise site-hydrophobicity modulation, which effectively prevented electrode flooding and stabilized the three-phase interface, thereby achieving a high FE_CO_ of 97.7% (at −1.0 V vs. RHE) and maintaining >90% FE_CO_ over a wide potential window from −0.8 to −1.2 V vs. RHE in CO_2_RR. Similarly, Gong et al. [71] covalently grafted amino-substituted NiPc (NiPc-NH_2_) and ODA onto CNTs, as shown in Figure 6, creating a superhydrophobic interface (NiPc-NH_2_/CNT-SHP) for acidic CO_2_RR. This robust covalent architecture not only immobilized single-molecule heterojunctions but also utilized *In Situ*-generated amino cations (-NH_3_^+^) to repel hydronium ions and hydrophobic chains to disrupt the interfacial water network, achieving nearly 90% FE_CO_ over a broad current density range (100–300 mA cm^−2^) and maintaining >80% FE_CO_ for over 200 h in an integrated porous solid electrolyte reactor. Xu et al. [72] covalently anchored carboxyl-functionalized CoPc (CoPc-COOH) onto amino-functionalized CNT (CNT-NH_2_) via amidation. This “click chemistry” approach not only improved molecular dispersion but also induced charge transfer from the Co center to the CNT matrix, facilitating the generation of active Co(I) sites at lower potentials. The resulting CoPc-COOH/CNT-NH_2_ hybrid achieved a CO partial current density of 22.4 mA cm^−2^ with 91% FE_CO_ at -0.88 V vs. RHE for CO_2_-to-CO conversion. However, despite the robustness of this covalent linkage, the resultant amide bonds remain susceptible to hydrolysis under extreme acidic or alkaline electrolyte conditions, posing a latent risk of CoPc molecular leaching that could compromise the long-term operational stability of the catalyst. Liu et al. [73] directly immobilized NiPc onto CNTs via a robust C-C covalent linkage (Ni-CNT-CC), formed by converting the four peripheral amino groups of 2,9,16,23-tetra(amino)-NiPc into diazonium salts followed by elimination through a free-radical reaction. This architecture facilitated efficient charge transfer and achieved >90% FE_CO_ across a wide potential window in 0.5 M KHCO_3_, maintaining stable operation for over 100 h without activity decay. Nevertheless, despite the excellent durability conferred by the C–C linkage, the synthetic complexity associated with peripheral group conversion and radical reactions renders large-scale production impractical.

### 3.3. Geometric and Spatial Engineering

CNTs extend beyond their roles as conductive, dispersive scaffolds and can serve as active structural templates. Their inherent physical attributes, including curvature, atomic-scale defects, and one-dimensional cavities, can impose mechanical strain or offer specific anchoring sites to supported MPcs. These strategies allow induction of geometric distortion and symmetry breaking in the catalysts, as well as the creation of unique local microenvironments, thereby unlocking novel catalytic functionalities. A prime example of this effect is the switch in the CO_2_RR pathway for CoPc. While CoPc on conventional flat supports typically produces CO via a two-electron pathway [10,11], its support on CNTs enables the selective six-electron reduction in CO_2_ to methanol (CH_3_OH) [74,75].

**Figure 7:**
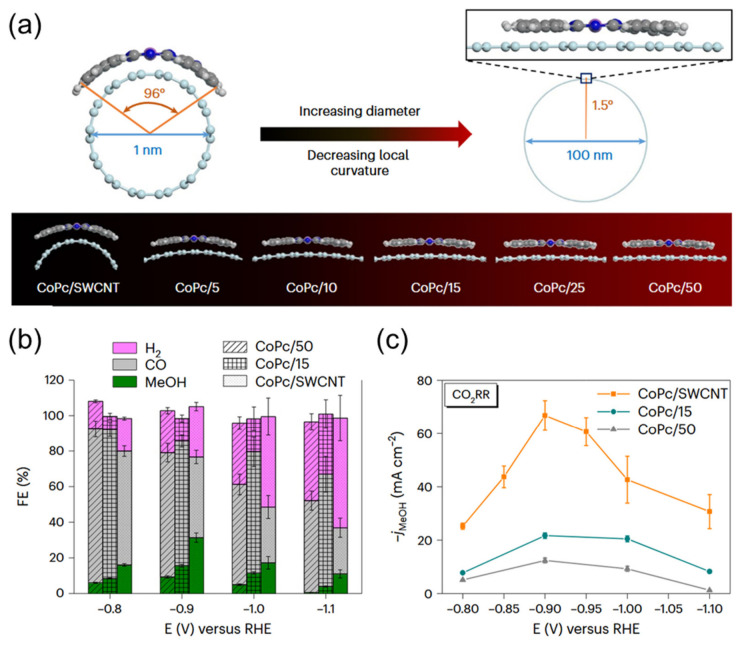
(**a**) Illustration of the structural distortion of CoPc molecules adsorbed on CNTs with varying diameters, showing the transition from a highly bent configuration on single−walled CNTs (CoPc/SWCNT) to a progressively flattened geometry on larger−diameter CNTs, denoted as CoPc/x(x represents the radius of the CNT in nm). (**b**) FE_CH3OH_ and (**c**) j_MeOH_ for CO_2_RR in a flow cell containing 0.1 M KOH + 3 M KCl. Copyright 2023 by Springer Nature [77].

The transition from CO to methanol production is intrinsically linked to the dispersion of CoPc on the CNTs surface. Wu et al. [76] first reported that CoPc achieves the selective six-electron electroreduction of CO_2_ to methanol when molecularly dispersed on CNTs, whereas aggregated CoPc particles only produce CO and H_2_. Their key discovery was that a high degree of molecular dispersion of CoPc on the conductive CNT surface is a critical prerequisite, enabling efficient electron transfer to drive a unique “domino” reduction cascade via a *CO intermediate. This seminal work established the CoPc/CNT system as a platform for deep CO_2_ reduction and highlighted the defining role of catalyst dispersion, paving the way for subsequent studies to unravel the specific structural influences of the CNT supports. While this seminal work emphasized the importance of molecular dispersion, the critical role of the CNTs’ intrinsic structure remained underestimated until later studies shifted the focus to the support’s morphology. Subsequent studies have definitively established that the curvature-induced strain from SWCNT supports is the key structural factor unlocking this alternative reaction pathway. Su et al. [77] systematically demonstrated that CoPc supported on SWCNTs undergoes significant molecular bending, as it is shown in Figure 7, which strengthens the binding of the *CO intermediate to the Co–N_4_ site. This enhanced adsorption, driven by the strong molecule–support interaction, prevents *CO desorption and enables its further hydrogenation, allowing CoPc/SWCNT to achieve a methanol partial current density 66.8 mA cm^−2^ with 31.3% FE_CH3OH_ in a flow cell, far surpassing the performance on larger-diameter MWCNTs. Xin et al. [78] provided a direct comparative study, revealing that while the curved configuration of Tetraamino CoPc (CoTAPc) on SWCNTs promotes favorable *CO hydrogenation for methanol production (with a FE_CH3OH_ up to 52%), the identical strained geometry applied to FePc leads to excessively strong *CO adsorption and rapid catalyst poisoning, decreasing its TOF by >50%. This contrast underscores that the impact of curvature strain is neither inherently positive nor negative but is critically determined by how it modifies the electronic structure and intermediate binding energetics specific to each metal center.

The CoPc molecular distortion induced by CNTs, and its direct link to methanol production, have been confirmed by advanced *In Situ* spectroscopic techniques (shown in Table 2). Ren et al. [79] used extended X-ray absorption fine structure analysis to demonstrate that anchoring CoPc on MWCNTs shortens the Co–C bond distance in the second coordination shell from 2.44 Å to 2.35 Å. This measurable structural distortion is proposed to generate a *CO intermediate with a weakened C–O bond during electrocatalysis, lowering its protonation barrier and enabling a high FE_CH3OH_ of 65% at 30 mA cm^−2^. Critically, this geometric change is accompanied by a reduction in molecular symmetry and enhanced electronic coupling with the support. Rooney et al. [80] employed *In Situ* X-ray absorption spectroscopy to reveal that molecularly dispersed CoPc on CNT surfaces undergoes a symmetry reduction from D_4_h to C_4_v. The observed spectral changes indicate both this symmetry-breaking and a strong electronic interaction between CoPc and the CNT. This synergistic modulation of the catalyst’s geometry and electronic structure is identified as the key to enabling efficient CO_2_-to-methanol conversion, achieving a FE_CH3OH_ of 40% at −0.95 V vs. RHE. Furthermore, Hutchison et al. [81] showed via DFT calculations that π-stacking of CoPc on a graphitic surface (modeling CNTs) modifies the catalyst’s electronic structure, facilitating key redox steps and altering proton-coupled electron transfer pathways for CO_2_-to-methanol conversion. This confirms that the CNTs actively participate electronically, and the overall catalytic enhancement is a concerted effect of geometric and electronic modulation. Liu et al. [82] further demonstrated using superconducting quantum interference device (SQUID) magnetometry that CNTs with diameters < 3 nm induce local symmetry distortion and a reduction in the crystal-field splitting energy (Δoct), triggering a spontaneous transition of Co^2+^ centers from low-spin to high-spin states. SQUID measurements directly confirmed this electronic reconfiguration through enhanced magnetic moments, establishing a direct correlation between CNT curvature-induced strain and spin-state modulation. Mechanistically, the high-spin state weakens N=O bonding while stabilizing bent *NO intermediates, enabling exceptional nitric oxide reduction (NORR) to NH_3_ with a 91.5% FE_NH3_ at −0.5 V vs. RHE. While these studies establish that CNTs actively modulate both geometry and spin states, truly elucidating the underlying mechanisms requires more advanced *operando* spectroscopies capable of resolving dynamic structural evolution under realistic reaction conditions.

While detrimental for FePc’s CO_2_RR performance, curvature-induced strain can be strategically harnessed to enhance its performance for ORR by tailoring the electronic structure to optimize intermediate binding. Specifically, Musgrave et al. [83] loaded FePc onto SWCNTs to induce molecular strain, contrasting it with the flat configuration on MWCNTs, and discovered that the curvature-induced strain elongated the Fe-N bonds to alter the rate-determining step from *O_2_ adsorption to *OH desorption, thereby accelerating electron transfer and delivering a superior half-wave potential of 0.952 V vs. RHE (compared to 0.879 V for MWCNTs) with a lower Tafel slope of 35.7 mV dec^−1^ for the ORR. Extending this curvature-tuning strategy, Jie et al. [84] anchored FePc on a curved-graphdiyne/CNT hybrid, where the CNT serves as a curvature template and electron-donor layer that bends the cGDY overlayer and enhances the electron density of C_sp_–C_sp_ bonds, thereby strengthening the cation–π coupling with FePc. X-ray absorption spectroscopy revealed a symmetry reduction in FePc from D_4_h to C_4_v upon loading, and the resulting FePc/cGDY/CNT catalyst delivered a high half-wave potential of 0.905 V and a low Tafel slope of 31.7 mV dec^−1^ in alkaline ORR. The curvature-tuning strategy is equally applicable to catalysts derived from MPcs precursors. Xia et al. [85] reported that a CuPc-derived catalyst supported on CNTs enables continuous tuning of the ORR pathway via curvature engineering, where high curvature directs the Cu-N_4_ sites toward efficient 4e^−^ reduction with Pt/C-comparable activity, while low curvature switches the selectivity to the 2e^−^ pathway, achieving a high FE_H2O2_ of 96.4% at 200 mA cm^−2^.

In addition to curvature, atomic-scale defects on CNTs serve as high-affinity, specific anchoring sites for MPcs, offering another dimension for structural control. This localized anchoring triggers a geometric distortion of the MPcs’ macrocycle, which perturbs the symmetry of the metal-N_4_ center and optimizes the electronic structure for enhanced intermediate binding. For instance, Yang et al. [86] demonstrated that NiPc preferentially anchors at CNT defect sites rather than adsorbing uniformly via π–π stacking. By optimizing defect density via thermal graphitization, they tuned the resulting structural distortion of NiPc, achieving an exceptional FE_CO_ of near 100% in CO_2_RR and effectively suppressing HER. They [87] also found that the interaction between CoPc and defects on the CNT, under applied cathodic potential, induces an out-of-plane distortion of the planar Co-N_4_ center; this structural change transforms the *CO binding configuration from linear to bridged, thereby unlocking efficient methanol production with a partial current density exceeding 150 mA cm^−2^. Mei et al. [88] revealed that atomic-scale defects on CNTs serve as proton-feeding sites by lowering the energy barrier for water dissociation, thereby accelerating proton transfer to the CoPc active centers. These findings highlight the critical role of CNT supports in enabling potential-driven structural distortion and defect-driven water activation for enhanced electrocatalysis.

**Figure 8:**
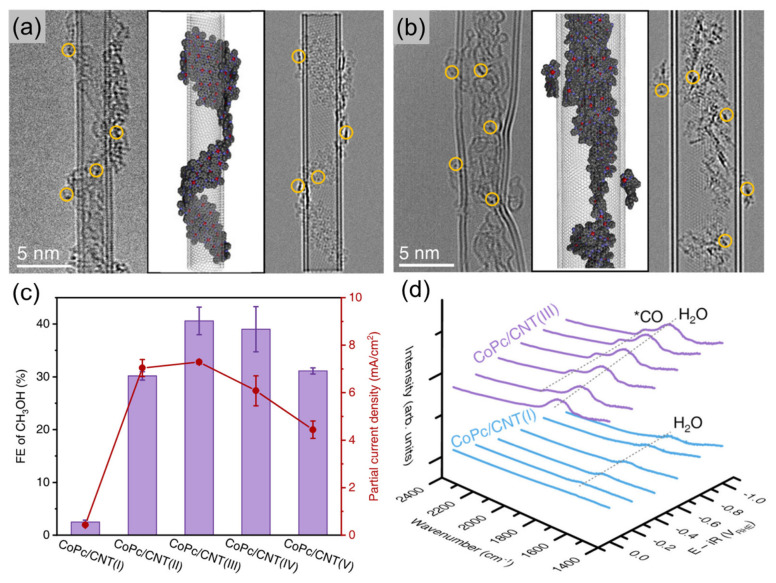
(**a**,**b**) HR–TEM images, structural models, and simulated TEM images of CoPc/CNT(I) (CoPc outside) and CoPc/CNT(III) (CoPc inside). Yellow circles highlight Co atoms. (**c**) The maximum FE_CH3OH_ and methanol partial current density for CoPc/CNTs(I–V) for CO_2_RR, measured in 0.1 M KHCO_3_. (**d**) Operando ATR–SEIRAS spectra of CoPc/CNT(I) and (III) at various potentials, indicating weak *CO adsorption on CoPc/CNT(I). Copyright 2025 by Springer Nature [89].

Beyond supporting MPc molecules on the external surface, encapsulation within the inner cavity of carbon nanotubes represents a distinct structural engineering strategy that leverages a unique nanoconfined environment [24]. Shi et al. [89] found that the catalytic pathway for CO_2_RR diverges dramatically based on the location of CoPc: molecules encapsulated inside the CNTs selectively produced methanol, while those located on the exterior surface primarily generated CO (Shown in Figure 8). This performance switch is attributed to the synergistic effects of the confined space. The nanocavity restricts the free diffusion of CO intermediates, leading to their local accumulation and providing a high concentration for further reduction. Concurrently, the spatial constraints imposed by the cavity wall induce structural deformation in the CoPc molecule, which enhances the adsorption of the *CO intermediate. These effects steer the reaction pathway toward the deep reduction to methanol, achieving a FE_CH3OH_ of 41% and a partial current density of 7.3 mA cm^−2^ at −0.96 V vs. RHE. Nevertheless, while this nanoconfinement effectively steers the reaction toward deep reduction, the inherently slow mass transport within the narrow cavities limits the partial current density of CH_3_OH, resulting in negligible methanol yields even in flow-cell configurations with enhanced mass transfer; however, this finding is retrospectively significant as it plausibly explains why early studies using CoPc/MWCNTs often neglect to detect CH_3_OH, overlooking the potential of internally confined sites in favor of the more dominant CO production on external surfaces. However, this interpretation overlooks the critical role of the curvature sign: the concave inner wall and convex outer wall impose fundamentally distinct spatial electronic modulations on the CoPc center, an aspect systematically decoupled and clarified by Pan et al. [90] through their matched convex/concave FePc model systems. They systematically engineered exclusive convex vs. concave curvature environments for FePc by constructing matched model supports—inverse cylindrical mesoporous carbon (convex-FePc) and its cylindrical mesopore counterpart (concave-FePc)—to decouple wall geometry from dispersion effects. They demonstrated that convex-FePc favors the 4e^−^ ORR, whereas concave-FePc delivers >80% selectivity to H_2_O_2_ (peaking at ~82.8%), and identified the governing mechanism as a curvature-sign–controlled spatial electronic effect: the confined concave curvature suppresses Fe d_z2_–O p orbital overlap with *OOH, weakening *OOH adsorption and steering the pathway toward 2e^−^ O_2_-to-H_2_O_2_, while the spatially open convex curvature enhances that d_z2_-mediated coupling to promote O–O breakage and 4e^−^ reduction.

Building on the foundational principles of structural engineering, diverse material design strategies are employed. Among these, constructing spatial dual-site architectures is a key approach, which involves engineering synergistic active sites for enhanced catalytic performance. Li et al. [91] demonstrated that co-loading CoPc-NH_2_ and NiPc-OCH_3_ molecules on MWCNTs creates a dual-site cascade catalyst, where the CNT support enables efficient molecular-scale CO spillover from Ni-N_4_ to Co-N_4_ sites, boosting the FE_CH3OH_ to 50% with a partial current density of 150 mA cm^−2^ in CO_2_RR. Beyond cascade reactions, constructing interfacial heterostructured diatomic sites offers another promising avenue; Chen et al. [92] developed a FePc/CoPc co-loaded CNT catalyst (FeCo-MHs) via a pyrolysis-free, specific-adsorption strategy. They identified well-defined FeCo “molecular heterostructures” where CoPc partially overlapped FePc, a configuration unambiguously confirmed by *In Situ* rotation AC-HAADF-STEM, which delivered exceptional ORR activity with a half-wave potential of 0.95 V and high stability. Another distinctive strategy involves modifying the carbon nanotube cavity. Zhu et al. [93] designed a FePc catalyst on a polyoxometalate-encapsulated CNT (FePc-{PW12}@CNT), where the internal {PW12} clusters strained the SWCNT and the external nanotube curvature cooperatively induced biaxial strain on the FePc molecule. This dual internal/external structural modulation broke the molecular symmetry, enabling superior ORR activity with a half-wave potential of 0.90 V, a Tafel slope of 30.9 mV dec^−1^.

Extending structural engineering from molecular-scale distortion to mesoscale architecture assembly, the construction of MPcs-based nanocomposites via molecular self-assembly represents a synergistic and scalable strategy. This approach mitigates molecular stacking while ensuring accessible active sites, efficient charge transport, and enhanced structural stability [94,95]. For instance, Li et al. [96] synthesized tetrafluoro-substituted CoPc (CoF_4_Pc) that forms ultra-small nanorods (length < 30 nm) highly dispersed on the surface of carbon nanotubes, akin to “lanterns hanging on a string.” This specific architecture prevents molecular aggregation and facilitates rapid charge transfer, enabling the CoF_4_Pc@CNT composite to achieve a high CO current density of 29.0 mA cm^−2^ with 91.8% FE_CO_ in CO_2_RR. Wang et al. [97] fabricated crystalline FePc nanorods via precipitation and decorated them on multi-walled carbon nanotubes, creating a similar architecture. This well-exposed and conductive structure enabled the N-FePc@CNT composite to achieve a 94% FE_NH3_ and a partial current density of ~75 mA cm^−2^ in NO_3_RR. Liang et al. [98] synthesized CoPc into extended nanorods and wrapped them with carboxylated multi-walled CNT (CNT-COOH), forming a “vine-like” conductive network that maximizes the interfacial contact to accelerate electrocatalytic kinetics. This intertwined architecture demonstrated exceptional stability, maintaining operation for 90 h without performance degradation while delivering high CO selectivity with a FE_CO_ exceeding 95.5%. The key parameters are shown in Table 3. These works highlight that self-assembled MPcs nanostructures on CNTs can effectively balance molecular dispersion and charge transport, leading to enhanced electrocatalytic performance.

## 4. Conclusions and Outlook

In summary, this review has systematically delineated the multifaceted role of carbon nanotubes (CNTs) in advancing metal phthalocyanine (MPc)-based electrocatalysis. Far from being inert supports, CNTs actively participate in the catalytic process by serving as conductive highways, structural templates, and electronic modulators. Through the sophisticated interplay of dispersion engineering, chemical bonding (axial coordination and covalent grafting), and geometric spatial control (curvature-induced strain and nanoconfinement), the MPc/CNT interface has been precisely tailored to deliver exceptional activity, selectivity, and stability across a wide range of sustainable energy conversion and environmental remediation reactions.

Looking forward, to propel this field from fundamental understanding towards practical implementation, future research should be directed along the following strategic trajectories:

First, elevating MPc/CNT to a benchmark platform for mechanistic decoding: Given the molecular precision of the M–N_4_ active sites and the tunability of the CNT environment, these hybrids constitute an ideal model system for establishing universal design principles in molecular catalysis. Future efforts must prioritize the application of advanced synchrotron-based X-ray techniques and high-level theoretical simulations under *operando* conditions. This will enable precise correlation of dynamic structural evolution—such as spin-state transitions, axial–ligand exchange, and molecular distortion—with catalytic turnover frequency, thereby moving beyond trial-and-error towards predictive catalyst design.

Second, expanding the catalytic repertoire to address industrial imperatives: While current research has focused on model reactions like CO_2_RR and ORR, there is an urgent need to leverage the robustness of MPc/CNT electrocatalysts to tackle more complex and industrially relevant transformations. This includes the electrosynthesis of high-value organic intermediates and the selective functionalization of inert small molecules. By rationally designing dual-site or multi-metallic configurations on CNT scaffolds, researchers can potentially overcome the limitations of single-site catalysts and facilitate intricate cascade reactions that are currently unattainable with conventional materials.

Third, engineering for industrial integration and system-level synergy: Achieving laboratory-scale performance is insufficient; the ultimate goal is deployment in zero-gap membrane electrode assemblies (MEAs) operating at ampere-level current densities. Future work must therefore focus on the macroscale engineering of the electrode architecture, optimizing the three-phase boundary to ensure efficient mass transport and ionic conduction while mitigating competitive side reactions. Furthermore, integrating these CNT-based electrocatalysts with intermittent renewable energy sources and developing automated, continuous-flow production systems will be pivotal to establishing economically viable and carbon-neutral chemical manufacturing processes.

## Figures and Tables

**Table 1:** Comparison of interfacial interaction modes for MPc/CNT electrocatalysts.

Design Strategy	Binding Force	Stability	Tuning Capability	Synthetic Complexity
Physical Dispersion	Noncovalent interactions (mainly π–π stacking)	Medium (potential risk of detachment)	Low (minimal impact)	Simple (widely adopted)
Axial Coordination	Metal-ligand coordination bonds	Strong (effectively suppresses metal site dissolution)	High (direct modulation of metal center)	Moderate (requires functionalization of MPcs or CNTs)
Covalent Grafting	Organic covalent bonds	Strong (effectively suppresses MPcs molecular detachment)	Medium (via linker groups)	High (requires precise design of bonding schemes)

**Table 2:** Representative characterization techniques for elucidating support-effect mechanisms in MPc/CNT electrocatalysts.

Characterization Technique	Key Information Obtained	Application in MPc/CNT	Evidentiary Status
X-ray Absorption Spectroscopy (XAS/XAFS)	Local coordination environment, oxidation state, bond length	Directly observes metal coordination changes induced by curvature/defects	Most direct and reliable
Electron Paramagnetic Resonance Spectroscopy (EPR)	Unpaired electron density	Confirms increased spin states and disruption of planar symmetry	Direct electronic evidence
X-ray Photoelectron Spectroscopy (XPS)	Surface elemental composition, valence state	Widely used to infer spin states and charge transfer at the metal center	Indirect inference
*In Situ* Infrared Spectroscopy (*IR*)	Adsorption configuration of intermediates	Captures reaction intermediates to map reaction pathways	Direct experimental probe
*Operando* Raman/*In Situ* XAS	Real-time structural evolution	Monitors catalyst stability and structural changes under working conditions	Gold standard for dynamic proof
Density Functional Theory (DFT)	Electronic structure, energy barriers, reaction pathways	Reveals possible mechanisms of distortion or confinement effects	Theoretical support

**Table 3:** Comparative summary of MPc/CNT-based electrocatalysts: engineering strategies, performance metrics, and stability.

MPcs Type	CNTs Type	Design Strategy	Reaction	Electrolyte	Cell	FE (%)	Partial Current Density (mA cm^−2^)	TOF (s^−1^)	Stability Test	Ref.
CuPc	MWCNTs (D: 10–25 nm; L: 10 μm)	Physical dispersion	NO_3_RR	1 M KOH + 1 M KNO_3_	H-cell	98	980	0.83	30 h (~300 mA cm^−2^)	[25]
					Flow cell	98.1	393	-		
CoPc						67.1	214			
			NO_2_RR	1 M KOH + 1 M KNO_2_		98	466			
CoPc	MWCNTs		ORR	1 M KPi	Flow cell	88.7	~200	0.6	-	[26]
CoPc	MWCNTs (D: 10–15 nm)		C_2_H_4_Cl_2_ to C_2_H_4_	0.1 M KHCO_3_ + 87 mM C_2_H_4_Cl_2_	H-cell	100	11	30	6 h (Electrofiltration experiment)	[27]
CoPc	-		C_2_H_4_ to C_2_H_6_O_2_	0.1 M NaClO_4_	H-cell	23.2	5.8	0.03	20 h	[28]
CoPc	MWCNTs		C-N coupled to CH_3_CHNOH	0.5 M PBS + 10 mM Aldehyde + 100 mM KNO_2_	H-cell	75	8.9	1.3	-	[29]
				0.2 M KHCO_3_ + 10 mM Aldehyde + 10 mM KNO_2_		44	2.6	0.43		
			C-N coupled to pyruvicoxime	0.5 M PBS + 10 mM Pyruvic acid + 100 mM KNO_2_		67	6.1	0.90		
CoPc	MWCNTs		C-S coupled to HMS	CO_2_-saturated; 100 mM Na_2_SO_4_ + 25 mM Na_2_SO_3_	H-cell	25.7	4.9	-	5 h	[30]
CoPc	MWCNTs		CO_2_RR to CO	0.05 M H_2_SO_4_	MEA	35	18	-	13 h	[31]
CoPc	MWCNTs (D: 10–25 nm; L: 10 μm)	Modification on CNTs	NO_3_RR	0.5 M KOH + 0.04 M KNO_3_	H-cell	70	8.4	-	-	[32]
	Oxidized MWCNTs					28	12			
CoPc	O-groups-functionalized MWCNTs		ORR	1 M Na_2_SO_4_	Flow cell	92	300	56	100 h	[33]
VOPc	GCNT-OH		ORR	0.5 M H_2_SO_4_ + 0.1 M K_2_SO_4_	Flow cell	92	3500	~910	40 h (500 mA cm^−2^)	[34]
CoPc2	MWCNTs (D: 6–9 nm; L: 5 μm)	Modification on MPcs	CO_2_RR to CO	0.5 M NaHCO_3_	H-cell	93	18.1	6.8	-	[35]
				1 M KOH	Flow cell	94	165	3.9	3 h (120 mA cm^−2^)	
NiPc-OMe	MWCNTs (D: 10–25 nm; L: 10 μm)		CO_2_RR to CO	0.5 M KHCO_3_	H-cell	100	14.5	2.9	-	[36]
				1 M KHCO_3_	Flow cell	99.1	400	-	40 h (150 mA cm^−2^)	
				0.1 M H_2_SO_4_ + 0.4 M K_2_SO_4_		98		-	12 h (100 mA cm^−2^)	[37]
Sn(OH)_2_Pc-8F	MWCNTs		CO_2_RR to Formate	1 M KOH	Flow cell	80.8	600	81	15 h (400 mA cm^−2^)	[38]
				1 M KHCO_3_	MEA	90	200	-	200 h (100 mA cm^−2^)	
FePc-NH_2_	MWCNTs (D: 8 nm)	Modification on MPcs/Hydrophobicity regulation	NO_3_RR	0.1 M NaOH + 0.1 M KNO_3_	H-cell	69	83.9	-	-	[39]
						94.1	22.9	145.2	6 h	
CoPc-DEG	CNT@CC	Modification on MPcs/Electrode design	HER	1 M KOH	H-cell	-	1000	-	25 h	[41]
CoPc	AD-PTFE-CNT	Hydrophobicity regulation	CO_2_RR to CO	1 M KOH	MEA	95	190	41.3	3 h (100 mA cm^−2^)	[42]
					Flow cell	85	170	74.3	-	
CoPc-TC	MWCNTs	Modification on MPcs/Hydrophobicity regulation	CO_2_RR to CO	0.5 M KHCO_3_	H-cell	95.5	23.7	29.3	8 h	[43]
					MEA	96	96	-	60 h (50 mA cm^−2^)	
CoPc	CNT@CC	Device design	CO_2_RR to CO	3 M KHCO_3_	Cross-flow BCE	96.2	289	-	13 h (100 mA cm^−2^)	[44]
CoPc	-	Loading amount control	CO_2_RR to CO	1 M KOH	Flow cell	95	190	83.9	-	[45]
				0.1 M KHCO_3_	MEA	93	186	-	40 h (50 mA cm^−2^)	
ZnPc	MWCNTs		NO_3_RR to NH_2_OH	1 M KOH + 1 M KNO_3_	H-cell	53	270	7.5	-	[46]
NiPc-(OH)_6_ (DCNFO)	1@CNT (22.1)	Auxiliary hydrogen bonds	CO_2_RR to CO	1 M KOH	Flow cell	98	620	-	40 h (230 mA cm^−2^)	[47]
CoPc (1⊃K^+^)	MWCNTs	Reinforced electrostatic attraction	CO_2_RR to CO	0.5 M KHCO_3_	H-cell	98	38	111	14 h (27 mA cm^−2^)	[48]
SnPc	MWCNTs-OH	Axial coordination	CO_2_RR to formate	1 M KOH	Flow cell	89.4	74.8	-	8 h	[51]
CoPc	Oxidized SWCNTs		ORR	0.1 M K_2_SO_4_	H-cell	83	16	-	75 h	[52]
FePc	CNTs-OH		CO_2_RR to CO	1 M KOH	Flow cell	95	111	14.7	12 h	[53]
CoPc	MWCNTs-OH		CO_2_RR to CH_3_OH	0.5 M KHCO_3_	H-cell	32	18.3	2.58	12 h	[54]
CoPc	MWCNTs-NH_2_		CO_2_RR to CO	1 M KOH	Flow cell	100	225	-	100 h	[56]
CoPc	N-CNTs (L: 15–30 μm)		CO_2_RR to CO	1 M KOH	Flow cell	95.1	761	-	60 h (100 mA cm^−2^)	[57]
CoPc	MWCNTs-Py (D: 8 nm)		CO_2_RR to CO	0.5 M NaHCO_3_	H-cell	98	5	34.5	-	[58]
CoPc	MWCNTs-Cy (D: 10–20 nm)		CO_2_RR to CO	0.5 M KHCO_3_	H-cell	93	26	3.61	12 h	[59]
	MWCNTs-Am					83	32	5.3		
	MWCNTs-Py					94	36	3.65		
FePc	CNTs-Py		NRR	0.1 M HCl	H-cell	22.2	0.026	-	50 h	[60]
FePc	CNTs-Py		NO_3_RR	1 M KOH + 0.1 M KNO_3_	H-cell	91	910-	-	120 h (1000 mA cm^−2^)	[61]
FePc	N-CNTs		C_2_H_2_ to C_2_H_4_	1 M KOH	Flow cell	86.7	434	117	110 h (100 mA cm^−2^)	[63]
NiPc	B-CNTs		CO_2_RR to CO	0.25 M K_2_SO_4_	Flow cell	98	80	-	37 h (40 mA cm^−2^)	[64]
P-CoPc	MWCNTs (D: 8–15 nm)		OER	1 M KOH	H-cell	-	10	0.071	18 h	[66]
CoTAMAPc	MWCNTs (D: 10–20 nm; L: 5–30 μm)	Covalent grafting	CO_2_RR to CO	1 M KOH	Flow cell	95.6	239	-	15 h (30 mA cm^−2^)	[69]
CoPc-NH_2_	MWCNTs-ODA		CO_2_RR to CO	0.5 M KHCO_3_	Flow cell	97.7	154.8	-	12 h	[70]
NiPc-NH_2_	MWCNTs-SHP		CO_2_RR to CO	0.1 M K_2_SO_4_ + 0.01 H_2_SO_4_	PSE	98	295.26	-	210 h (100 mA cm^−2^)	[71]
CoPc-COOH	MWCNTs-NH_2_		CO_2_RR to CO	0.5 M KHCO_3_	H-cell	91	22.4	-	48 h	[72]
NiTAPc	-		CO_2_RR to CO	0.5 M KHCO_3_	H-cell	90	83	27.8	100 h (17.5 mA cm^−2^)	[73]
CoPc	SWCNTs (D: 1 nm)	Curvature strain	CO_2_RR to CH_3_OH	0.5 M KHCO_3_	H-cell	15.5	1.6	-	-	[77]
				0.1 M KOH + 3 M KCl	Flow cell	31.3	66.8		10 h	
	MWCNTs (D: 50 nm)					9.3	9.3		-	
CoTAPc	SWCNTs (D: 1–2 nm)			0.5 M KHCO_3_	H-cell	51.5	6	3.2	10 h	[78]
	MWCNTs (D: >50 nm)					22	2.88	1.7	-	
FePc	SWCNTs (D: 1–2 nm)		CO_2_RR to CO			99	0.2	0.11		
	MWCNTs (D: >50 nm)					99	0.6	0.23		
CoPc	MWCNTs		CORR to CH_3_OH	0.25 M K_2_HPO_4_	MEA	65	19.5	-	-	[79]
CoPc	MWCNTs (D: 10–15 nm)			0.1 M KHCO_3_	H-cell	40	11	-	-	[80]
CoPc-NH_2_						20	6			
CoPc	SWCNTs (D: 1–2 nm)		NORR	0.5 M K_2_SO_4_	H-cell	91.5	21	-	20 h	[82]
	MWCNTs (D: 30–50 nm)					57	4		-	
NiTAPc	GCNT-H	Structural distortion	CO_2_RR to CO	0.5 M K_2_SO_4_ (pH = 2)	MEA	99.9	400	292	-	[86]
	GCNT-L					99.9		648		
CoTAPc	GCNT		CORR to CH_3_OH	1 M KHCO_3_	Flow cell	52.6	105.2	-	-	[87]
						37.6	150.4			
			CO_2_RR to CH_3_OH			12.8	102.4			
				1 M KOH		10.5	84			
				0.5 M K_2_SO_4_ (pH = 2)		12.1	96.8			
CoPc	SWCNTs (ID: 1–2 nm)	Nanoconfinement	CO_2_RR to CH_3_OH	0.1 M KHCO_3_	H-cell	3	0.5	0.03	-	[89]
	SWCNTs (ID: 3 nm)					41	7.3	0.35		
CoPc-NH_2_ + NiPc-OCH_3_	MWCNTs	Dual-site architecture	CO_2_RR to CH_3_OH	0.1 M KHCO_3_	H-cell	43.4	15.6	-	11.5 h	[91]
				0.3 M KHCO_3_	Flow cell	50	150		5 h	
FePc	MWCNTs-COOH (D: 4–6 nm; L: 10–20 μm)	Nanocomposite design	NO_3_RR	1 M NaOH + 0.1 M NaNO_3_	H-cell	94	52.6	0.3	5 h	[97]
CoPc	MWCNTs-COOH (D: 5–15 nm; L: 10–30 μm)		CO_2_RR to CO	1 M NaHCO_3_	Desalination Cell	95.5	38.2	-	90 h	[98]

## Data Availability

No new data were created or analyzed in this study. Data sharing is not applicable to this article.

## Abbreviations

The following abbreviations are used in this manuscript:

MPc	Metal phthalocyanine
CoPc	Cobalt phthalocyanine
NiPc	Nickel phthalocyanine
CuPc	Copper phthalocyanine
FePc	Iron phthalocyanine
ZnPc	Zinc phthalocyanine
SnPc	Tin phthalocyanine
CNTs	Carbon nanotubes
SWCNT	Single-walled nanotube
MWCNT	Multi-walled nanotube
FE	Faraday efficiency
TOF	Turnover frequency
CO_2_RR	Carbon dioxide reduction reaction
NO_3_RR	Nitrate reduction reaction
NORR	Nitric oxide reduction reaction
NRR	Nitrogen reduction reaction
HER	Hydrogen evolution reaction
ORR	Oxygen reduction reaction
MEA	Membrane electrode assembly
BEC	Bicarbonate electrolyzer
STEM	Scanning transmission electron microscopy
XPS	X-ray photoelectron spectroscopy
EDS	Energy dispersive spectrometer
XAS	X-ray absorption spectroscopy
XAFS	Extended X-ray absorption fine structure analysis
DFT	Density functional theory
IR	Infrared spectroscopy
EPR	Electron paramagnetic resonance
SQUID	Superconducting quantum interference device
